# Supplementary material for: Identification of Freezing-Responsive microRNAs and Their Targets in Chinese Jujube by Small RNA and Degradome Sequencing
Source: Int J Mol Sci. 2026 Sep 4;27(17):7912. doi: 10.3390/ijms27177912 (PMC13566890; doi:10.3390/ijms27177912)
Supplement: Supplementary file 1 [file ijms-27-07912-s001.zip › Supplementary Material S2/novel_miR_103.pdf]

Provisional ID	:	novel_miR_103
Score total	:	0.5
Score for star read(s)	:	-1.3
Score for read counts	:	0
Score for mfe	:	0.2
Score for randfold	:	1.6
Score for cons. seed	:	
Total read count	:	738
Mature read count	:	214
Loop read count	:	524
Star read count	:	0

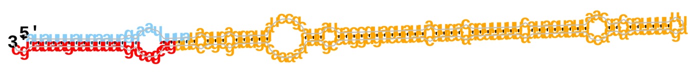

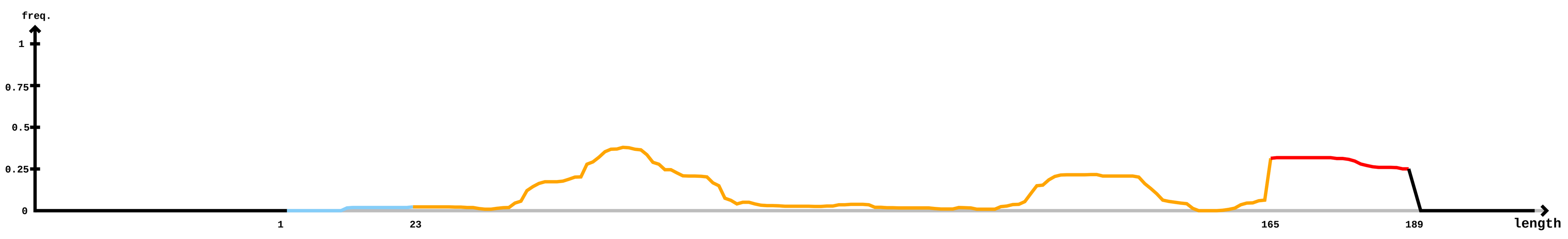

Star

Mature

```
5'- ggauagcaauacaaauacacacaugaauuuauacauguauauauuuaugaauggaaauuuuauagugaggaugauccgcaugaugaccguaguauuagugacgguuuuucauaguauuaacgacgauuuuuuagaaaaucaucaccaauacuauaaaaaacuaucaucaauacugugguccgcauaaaaaccguuugcgccauagacgaacuguauuuaugaauaugcgacuaacaauaaagaauacaau -3'
    ...................(((((........))))).(((((((((((((.(...(((((.(((.(((((......(((..(((((((((((.((((.(((((((.((((((((...((.((((((....)))))).))...)))))))).))))))).)))).)))))))))))..))).....)))))).))).)))))).....).))))))))))))))........................
```

read	reads	mm	sample
auggaaauuuuauagugC	1	1	LT-2
auggaaauuuuauUgugagg	2	1	LT-2
auggaaauuuuauagugaggaC	1	1	JCK-1
aCggaaauuuuauagugaggau	2	1	JCK-3
aCggaaauuuuauagugaggaug	1	1	JCK-2
aCggaaauuuuauagugaggaug	1	1	JCK-1
aCggaaauuuuauagugaggaug	1	1	JCK-3
auggaaauuAuauagugaggauga	3	1	JCK-3
auggaaauuuuauagugaggaugG	1	1	JCK-1
uggaaauuuuauagugaggaC	1	1	JCK-1
uggaaauuuuauagugaggaC	1	1	JCK-3
auagugaggaCgauccgca	2	1	JT-2
auagugaggaCgauccgca	1	1	JT-3
uccgcaugaAgaccguaguauu	1	1	LT-1
uccgcaugaAgaccguaguauu	2	1	LT-3
uccgcaugaAgaccguaguauua	1	1	LT-1
ccgcaugaAgaccguaguauu	1	1	LT-1
ccgcaugaAgaccguaguauuagu	1	1	LT-3
ccgcaugaAgaccguaguauuagu	1	1	LT-1
ccgcaugaAgaccguaguauuagu	1	1	JCK-2
cAcaugaugaccguaguauu	1	1	JCK-3
cgcaugaAgaccguaguauuag	1	1	LT-3
cgcaugaAgaccguaguauuagu	1	1	JCK-2
gcaugaAgaccguaguauuagu	1	1	LT-2
caugaAgaccguaguauu	3	1	LT-2
caugaAgaccguaguauua	1	1	JCK-2
caugaAgaccguaguauuag	5	1	LCK-3
caugaAgaccguaguauuag	2	1	LT-2
caugaAgaccguaguauuagu	2	1	LT-3
caugaAgaccguaguauuagug	2	1	LT-3
caugaAgaccguaguauuaguga	1	1	LT-1
caugaAgaccguaguauuagugac	2	1	JCK-3
caugaAgaccguaguauuagugac	2	1	JCK-2
caugaugaccguaguauuagugac	1	0	JCK-3

Star **Mature**

ggauagcaauacaaauacacacaugaauuuauacauguauauauuuaugaauggaaauuuuauagugaggaugauccgcaugaugaccguaguauuagugacgguuuuucauaguauuaacgacgauuuuuuagaaaaucaucaccaauacuauaaaaaacuaucaucaauacugugguccgcauaaaaaccguuugcgccauagacgaacuguauuuaugaauaugcgacuaacaauaaagaauacaau

augaAgaccguaguauuagug	2	1	JCK-2
augaAgaccguaguauuagug	1	1	LT-1
augaAgaccguaguauuagug	1	1	LT-3
augaAgaccguaguauuagug	4	1	JCK-3
augaAgaccguaguauuaguga	1	1	LT-1
ugaAgaccguaguauuagu	2	1	LT-3
ugaugaccguaguauuagug	1	0	JCK-3
ugaAgaccguaguauuagug	4	1	LT-3
ugaugaccguaguauuagug	1	0	JCK-2
ugaAgaccguaguauuagug	6	1	LT-2
ugaAgaccguaguauuagug	1	1	LT-1
ugaAgaccguaguauuaguga	5	1	JCK-1
ugaAgaccguaguauuaguga	11	1	JCK-2
ugaAgaccguaguauuaguga	8	1	LT-3
ugaugaccguaguauuaguga	1	0	JCK-3
ugaAgaccguaguauuaguga	8	1	JCK-3
ugaugaccguaguauuaguga	1	0	LT-1
ugaugaccguaguauuagugac	1	0	JCK-3
ugaAgaccguaguauuagugac	1	1	LT-3
ugaAgaccguaguauuagugacg	2	1	LT-2
gaAgaccguaguauuagug	1	1	LT-2
gaugaccguaguauuagugac	1	0	LT-3
gaAgaccguaguauuagugac	1	1	JCK-1
gaugaccguaguauuagugacA	1	1	LT-3
gaAgaccguaguauuagugacg	11	1	LCK-3
gaAgaccguaguauuagugacg	2	1	LT-3
gaAgaccguaguauuagugacg	2	1	LT-2
augacUguaguauuagugacggu	1	1	LT-1
augacUguaguauuagugacggu	2	1	JCK-2
augacUguaguauuagugacggu	9	1	LT-2
augacAguaguauuagugacgguu	1	1	JCK-3
augacAguaguauuagugacgguu	2	1	JCK-2
Agaccguaguauuagugacg	5	1	LCK-3
Agaccguaguauuagugacg	3	1	LT-2
cUguaguauuagugacgguu	3	1	LT-2
cguaguauuagugacgUu	1	1	LT-2
Uguaguauuagugacggu	2	1	LT-1
cguaguauuagugacAguu	1	1	JCK-3
cguaguauuagugacAguu	4	1	LT-2
cgGaguauuagugacgguu	1	1	JCK-2
guaguauuagugacgguu	2	0	LT-2
guaguauuagugacgguuu	1	0	LT-1
guaguauuagugacgguuuuu	2	0	LT-1
guaguauuagugacgguuuuucU	1	1	JCK-1
guaguauuagugacgguuuuucU	1	1	JCK-2
guaguauuagugacgguuuuucau	1	0	JCK-3
guaguauuagugacgguuuuucau	2	0	JCK-1
uagAauuagugacgguuuuucau	1	1	JCK-3
aguauuagugacgguuuuuc	1	0	LT-1
aguauuagugacgguuuuucU	1	1	LT-3
aguauuagugacgguuuuuca	1	0	LT-1
aguauuagugacgguuuuuca	5	0	JCK-2
aguauuagugacgguuuuuca	8	0	LT-2
aguauuagugacgguuuuuca	1	0	JCK-1
aguauuagugacggAuuuuca	1	1	LT-3
aguauuagugacgguuuuuca	2	0	JCK-3
aguauuagugacgguuuuucau	3	0	LT-1
aguauuagugacgguuuuucauG	8	1	JCK-2
aguauuagugacgguuuuucauG	3	1	JCK-3
aguauuagugacgguuuuucaGa	1	1	JCK-3
aguauuagugacgguuuuucauC	1	1	JCK-2
aguauuagugacgguuuuucaua	1	0	LT-3
aguauuagugacgguuuuuGaua	1	1	JCK-1
aguauuagugacgguuuuucaua	1	0	JCK-2
aguauuagugacgguuuuucauG	2	1	JCK-1
aguauuagugacgguuuuucaua	3	0	JCK-3
aguauuagugacgguuuuucauC	1	1	JCK-1
aguauuagugacgguuuuucaGa	1	1	JCK-2
aguUuuagugacgguuuuucaua	1	1	JCK-2
aguauuagugacgguuuuucaGa	1	1	LT-3

Star — Mature

ggauagcaauacaaauacacacaugaauuuauacauguauauauuuaugaauggaaauuuuauagugaggaugauccgcaugaugaccguaguauuagugacgguuuuucauaguauuaacgacgauuuuuuagaaaaucaucaccaauacuauaaaaaaacuaucaucaauacugugguccgcauaaaaaccguuugcgccauagacgaacuguauuuaugaauaugcgacuaacaauaaagaauacaau

aguauuagugacgguuuuucaGa	1	1	JCK-1
aguauuagugacgguuuuuGaua	2	1	JCK-3
aguauuagugacgguuuuucauag	2	0	JCK-3
aguauuagugacgguuuuucauag	4	0	JCK-1
aguauuagugacgguuuuucauag	3	0	JCK-2
guauuagugacggAuuuuc	4	1	LT-2
guauuagugacggAuuuuca	3	1	LCK-3
guauuagugacgguuuuucUu	1	1	JCK-2
guauuagugacgguuuuucauagu	1	0	JCK-3
guauuagugacgguuuuucauagu	1	0	JCK-1
guauuagugacgguuuuucauagu	1	0	JCK-2
uauuagugacgguuuuuc	1	0	LT-2
uauuagugacgguuuuuca	3	0	LT-2
uauuagugacgguuuuucau	3	0	LT-3
uauuagugacgguuuuucaua	3	0	JCK-2
uauuagugacgguuuuucaua	2	0	JCK-1
uauuagugacgguuuuucaua	5	0	JCK-3
uauuagugacgguuuuucaua	1	0	LT-3
uauuagugacgguuuuucaua	4	0	LT-2
auuagugacgguuuuuca	1	0	JCK-1
auuagugacgguuuuucau	1	0	LT-1
auuagugacgguuuuucau	1	0	LT-3
auuagugacgguuuuucau	1	0	JCK-2
auuagugacgguuuuucauG	1	1	JCK-2
auuagugacgguuuuucauG	1	1	JCK-1
auuagugacgguuuuucaua	1	0	JCK-1
auuagugacgguuuuucaua	2	0	JCK-2
auuagugacgguuuuucauG	1	1	LT-1
auuagugacgguuuuucaua	2	0	JCK-3
auuagugacgguuuuucauG	4	1	JCK-3
auuagugacgguuuuucaua	1	0	LT-3
auuagugacgguuuuucauU	2	1	LT-3
auuagugacgguuuAucauag	1	1	LT-3
auuagugacgguAuuucauagu	1	1	JCK-2
auuagugacgguAuuucauagu	3	1	JCK-3
auuagugacgguuuuucauaguG	2	1	JCK-1
uuagugacgguAuuucau	2	1	LT-3
uuagugacgguAuuucauagu	1	1	LT-1
uuagAgacgguuuuucauagu	2	1	LT-2
uuagugacgguuuuucauagu	3	0	JCK-3
uuagugacgguuuuucauagu	1	0	JCK-2
uuagugacgguAuuucauagu	1	1	LT-3
uuagugacgguuuuucauaguauu	1	0	LT-2
uuagugacgguuuuucauaguauu	1	0	JCK-3
uagugacgguuuuucaua	1	0	LT-3
agugacgguuuuucauagu	2	0	LT-3
agugacgguuuuucauagu	1	0	JCK-1
agugacgguuuuucauaguau	1	0	LT-1
agugacggAuuuucauaguauu	1	1	LT-3
agugacgguuuuucauaguauu	2	0	LCK-3
agugCcgguuuuucauaguauu	1	1	LCK-3
agugacgguuuuucauaguauua	3	0	LT-2
agugacgguuuuucauaguauua	1	0	JCK-2
agugacgguuuuucauaguauua	1	0	JCK-3
agugacgguuuuucauaguauuC	1	1	JCK-1
agugacgguuuuucauaguauuaU	1	1	JCK-2
agugacgguuuuucauaguauuaU	1	1	JCK-1
ugacgguCuuucauaguauua	1	1	LT-1
gacgguuuuucauagAau	1	1	LT-3
gacgguuuuucauaguauu	1	0	LT-3
gacgguuuuucauagAauu	1	1	LT-1
gacgguuuuuGauaguauu	1	1	LT-2
gacgguCuuucauaguauuaacga	1	1	LT-1
gacgguCuuucauaguauuaacga	1	1	JCK-1
acgguuuuucauaguauuaaGg	1	1	LT-2
cauaguauuaacgacgauuCu	1	1	LT-3
auaguauuGacgacgauu	1	1	LT-1
auaguauGaacgacgauuuu	2	1	LT-2
aguauGaacgacgauuuuuuagaaaa	2	1	LT-1
auuaacgacgauAuuuuagaaa	1	1	JCK-3

Star			
ggauagcaauacaaauacacacaugaauuuauacauguauauauuuaugaauggaaauuuuauagugaggaugauccgcaugaugaccguaguauuagugacgguuuuucauaguauuaacgacgauuuuuuagaaaaucaucaccaauacuauaaaaaacuaucaucaauacugugguccgcauaaaaaccguuugcgccauagacgaacuguauuuaugaauaugcgacuaacaauaaagaauacaau (Mature: gacgaacuguauuuaugaauaugc)			
uuaacgacgauuuuuuagaaaa	5	0	LCK-2
uuaacgacgauuuuuuagaaaa	5	0	LCK-1
uaacgacgauuGuuuagaaaauc	2	1	LT-2
aaGgacgauuuuuuagaaa	1	1	JCK-1
acgacgauuGuuuagaaaaucau	1	1	LT-3
uuagaaaaucaucaccaU	3	1	JCK-3
uuagaaaaucaucaccaau	2	0	LT-1
agaaaaucaucaccaauacua	1	0	JCK-3
agaaaaucaucaccaaCacuau	1	1	JCK-2
agaaaaucaucaccaauacuaua	1	0	LT-1
agaaaaucaucaccaauacuaua	1	0	LT-3
agaaaaucaucaccaUuacuaua	1	1	JCK-2
agaaaaucaucaccaauacuaua	1	0	JCK-2
aaaaCcaucaccaauacuaua	1	1	JCK-3
aaaaucaucaccaauacuaua	1	0	JCK-2
auaaaaaacuaucaucaauaUugu	2	1	JCK-3
auaaaaaacuaucaucaauaUugu	3	1	JCK-2
auaaaaaacuaucaucaauaUugu	2	1	JCK-1
acuaucaucaauacuguggucUgc	5	1	LT-1
acuaucaucaauacuguggucUgc	3	1	JCK-1
acuaucaucaauacuguggucUgc	4	1	JCK-2
cuaucaucaauacugugguccgc	2	0	JCK-3
cuaucaucaauacugugguccgc	1	0	JCK-2
uaucaucaauacugugguccg	1	0	JCK-2
uaucaucaauacugugguccg	1	0	JCK-1
uaucaucaauacugugguccgca	2	0	LT-2
uaucaucaauacugugguccgUa	1	1	LT-1
uaucaucaauacugugguccgcau	1	0	JCK-3
uaucaucaauacugugguccgcau	1	0	JCK-2
aucaucaauacugugguccgca	1	0	JCK-3
ucaucaauacugugguccgc	2	0	JCK-2
ucaucaauacugugguUcgc	2	1	LT-3
ucaucaauacugugguccgc	2	0	LT-1
ucaucaauacugugguccgca	1	0	JCK-1
ucaucaauacugugguccgca	1	0	JCK-3
ucaucaauacugugguccgca	1	0	JCK-2
ucaucaauacugugguUcgcaua	2	1	JCK-1
ucaucaauacugugguUcgcaua	1	1	JCK-2
ucaucaauacugugguccAcaua	1	1	LT-3
caucaauacugugguccg	3	0	LCK-3
caucaauacugugAuccgc	2	1	JCK-2
caucaauacugugguccgc	1	0	JCK-2
caucaauacugugAuccgc	1	1	JCK-3
caucaauacugugUuccgc	2	1	LT-2
caucaauacugugUuccgc	4	1	LCK-3
caucaauacugugguUcgca	1	1	JCK-3
caucaauacugugguUcgca	2	1	LT-3
caucaauacugugAuccgca	1	1	LT-1
caucaauacugugguccgca	1	0	LT-1
caucaauCcugugguccgca	1	1	LT-1
caucaauacugugguccgca	1	0	JT-1
caucaauacugugguUcgca	1	1	JCK-2
caucaauacugugAuccgca	2	1	LT-2
caucaauacugugguccgca	1	0	LT-3
caucaauacugugAuccgcaua	1	1	LT-1
caucaauacugugguccgcauC	1	1	JCK-3
caucaauacugugguccgcaua	4	0	LT-2
caucaauacugugguccgcauC	2	1	JCK-2
caucaauacugugguccgcauC	3	1	JCK-1
caucaauacugugguccgcaua	1	0	LT-3
caucaauacugugguUcgcaua	1	1	LT-1
caucaauacuguggucUgcauaa	1	1	LT-2
aucaauacugugguUcgca	5	1	LCK-3
aucaauacugugguccgcau	7	0	LCK-3
aucaauacugugAuccgcau	5	1	LCK-3
aucaauacugugguccgcau	8	0	LT-2
aucaauacugugguccgcaua	2	0	JCK-2
aucaauacugugguccgcaua	2	0	JCK-1
aucaauacugugguUcgcaua	3	1	JCK-2
aucaauacugugguUcgcaua	1	1	JCK-1

Star / Mature

ggauagcaauacaaauacacacaugaauuuauacauguau**auauuuaugaauggaaauuuua**uagugaggaugauccgcaugaugaccguaguauuagugacgguuuuucauaguauuaacgacgauuuuuuagaaaaucaucaccaauacuauaaaaaacuaucaucaauacugugguccgcauaaaaaccguuugcgccaua**gacgaacuguauuuaugaauaugc**gacuaacaauaaagaauacaau

.aucaauacugugguccgcaua.	1	0	JCK-3
.aucaauacugugguccgcauaaa.	1	0	LT-3
.aucaauacugugguccgcauaaaa.	1	0	JCK-3
.aucaauacugugguccgcauaaaa.	1	0	JCK-2
.ucGauacugugguccgcau.	3	1	LT-2
.caauacugugguccgcaCa.	2	1	LT-1
.caauacugugguccgcauaaaa.	2	0	LT-1
.caauacugugguccgcauaaaaa.	1	0	JCK-1
.caauacugugguccgcauaaaaac.	6	0	JCK-2
.caauacuguAguccgcauaaaaac.	1	1	LT-1
.caauacuguggucUgcauaaaaac.	1	1	JCK-2
.caauacugugguccgcauaaaaac.	2	0	JCK-1
.caauacugugguccgUauaaaaac.	1	1	JCK-3
.caauacugugguccgUauaaaaac.	2	1	JCK-2
.caauacuguggucUgcauaaaaac.	1	1	JCK-1
.cNauacugugguccgcauaaaaac.	1	1	JCK-2
.caauacugugguccgcauaaaaac.	5	0	JCK-3
.aauacuguggucUgcaua.	1	1	JCK-3
.aauacuguggucUgcaua.	1	1	JCK-1
.aauacugugguccgcauaa.	1	0	LT-1
.aauacuguggucUgcauaa.	1	1	JCK-1
.aauacugugguccgcauaa.	1	0	JCK-2
.aauacugugguccgcauaa.	2	0	JCK-3
.aauacugugguccgcauaaaaa.	1	0	JCK-2
.aauacugugguccgcauaaaaac.	1	0	JCK-2
.aauacuguggucUgcauaaaaacc.	2	1	JCK-3
.aauacugugguccgcauaaaaacc.	1	0	JCK-3
.aauacuguggucUgcauaaaaacc.	2	1	JCK-2
.aauacuguggucUgcauaaaaacc.	1	1	JCK-1
.aauacugugguccgcauaaaaacc.	1	0	JCK-2
.auacugugAuccgcauaaa.	1	1	LT-3
.auacugugguccgcaAaaa.	2	1	LT-3
.auacugugguccgcauaaaaa.	1	0	JCK-1
.auacugugAuccgcauaaaaacc.	2	1	JCK-3
.auacugugAuccgcauaaaaacc.	1	1	JCK-2
.uacugugguccgcauaaaaac.	1	0	LT-2
.guggucGgcauaaaaacc.	1	1	JCK-1
.gcgccauagacgaacuguauuuG.	2	1	LT-1
.cgccauagacgaacugua.	4	0	LCK-3
.gccauagacgaacuguauuuau.	6	0	LCK-3
.ccauagacgGacuguauu.	1	1	JCK-3
.ccauagacgGacuguauu.	1	1	JCK-1
.ccauagacgGacuguauu.	1	1	JCK-2
.cUauagacgaacuguauu.	1	1	LT-1
.ccauagacgGacuguauuu.	1	1	JCK-1
.ccauagacgGacuguauuu.	2	1	JCK-3
.ccauaAacgaacuguauuu.	1	1	LT-3
.ccauagacgGacuguauuu.	3	1	LT-3
.ccauagacgGacuguauuu.	1	1	LT-1
.ccauagacgaacuguauuuU.	1	1	LT-1
.ccauaAacgaacuguauuua.	3	1	LCK-3
.cauagacgGacuguauuua.	2	1	JCK-1
.cauagacgGacuguauuua.	1	1	JCK-3
.cauagacgaacuguauuuU.	1	1	LT-3
.cauagacgGacuguauuua.	1	1	JCK-2
.cauagacgGacuguauuua.	3	1	LT-3
.auagacgaacuguauuuaA.	1	1	LT-3
.uCgacgaacuguauuuaug.	1	1	LT-2
.uagacgaacuguauuuaug.	2	0	LT-1
.uagacgaacuguauuuaug.	3	0	LT-2
.uagacgaacuguauuuaugaauau.	1	0	JCK-2
.uagacgaacuguauuuaugaauau.	1	0	JCK-1
.uagacgaacuguauuuaugaauau.	2	0	JCK-3
.agacgaacuguauuuauga.	3	0	LCK-3
.gacgaacuguauuuaugaaua.	1	0	JCK-3
.gacgaacuguauuuaugaauau.	1	0	JCK-2
.gacgaacuguauuuaugaauau.	1	0	LT-1
.gacgaacuguauuuaugaauaugc.	11	0	LT-3
.gacgaacuguauuuaugaauaugc.	57	0	JCK-2
.gacgCacuguauuuaugaauaugc.	1	1	JCK-2

```
                                       Star                                                                                                                                                                            Mature
ggauagcaauacaaauacacacaugaauuuauacauguauauauuuaugaauggaaauuuuauagugaggaugauccgcaugaugaccguaguauuagugacgguuuuucauaguauuaacgacgauuuuuuagaaaaucaucaccaauacuauaaaaaacuaucaucaauacugugguccgcauaaaaaccguuugcgccauagacgaacuguauuuaugaauaugcgacuaacaauaaagaauacaau
............................................................................................................................................................................................................gacgaacuguauuuaugaauaugc......................   53   0   JCK-3
............................................................................................................................................................................................................gacgaacuguauuuaugaCuaugc......................   1    1   JCK-2
............................................................................................................................................................................................................gacgaacuguauuuaugaauaugc......................   37   0   JCK-1
............................................................................................................................................................................................................gacgaacuguauuuaugaauaugc......................   12   0   LCK-3
............................................................................................................................................................................................................gacgaacuguauuuaugaauaugc......................   23   0   LT-1
.............................................................................................................................................................................................................acgaacuguauuuaugaauaugc......................   1    0   LT-2
.............................................................................................................................................................................................................acgaacuguauuuaugaauaugc......................   2    0   JCK-2
```
